# Comparison of Two Different Sizes of Endotracheal Tracheal Tube for Postoperative Sore Throat in Breast Cancer Patients Undergoing Surgeries

**DOI:** 10.7759/cureus.12896

**Published:** 2021-01-25

**Authors:** Sadia Ali, Ahsun Khan, Allah Ditta Ashfaq

**Affiliations:** 1 Anesthesia and Critical Care, Shaukat Khanum Memorial Cancer Hospital and Research Centre, Lahore, PAK

**Keywords:** postoperative sore throat, breast surgery, sore throat, endotracheal tube

## Abstract

Objective

The purpose of this study was to compare two different sizes of an endotracheal tube (ETT), with inner diameters (ID) of 6.5 mm and 7.5 mm, for the frequency of postoperative sore throat in patients undergoing elective breast cancer surgery.

Methodology

This study was a randomized controlled trial conducted in the Shaukat Khanum Memorial Cancer Hospital and Research Center from December 3, 2016, to February 3, 2017. This study included 110 patients, 55 from each group, who were selected from the elective surgery list fulfilling the inclusion criteria. Group A patients were intubated with 6.5-mm ID ETTs and group B patients with 7.5-mm ID ETTs. All patients were carefully extubated in the operating room when fulfilling extubation criteria. Any concerns of sore throat were recorded 24 hours postoperatively. All relevant data were recorded on a pro forma.

Results

In this study, the mean age was 46.6 ± 13.2 years (range: 18-65 years). The mean body mass index (BMI) was 29.50 ± 07.12 kg/m^2^, with a minimum and maximum of 19 kg/m^2 ^and 38 kg/m^2^, respectively. The mean four-point scale was 2.98 ± 1.3, with a minimum and maximum of 1.0 and 4.0, respectively. Of the 110 patients, 47 patients reported a sore throat at 24 hours after surgery, whereas 63 patients did not report a sore throat. In this study, 14 patients in group A were diagnosed with a postoperative sore throat, whereas 33 patients in group B were diagnosed with a sore throat at 24 hours. A chi-square test was significant, and a poststratification chi-square test was applied to compare sore throat at 24 hours postoperative between the groups with respect to age and BMI.

Conclusions

ETT sizes produced a significant difference in the frequency of postoperative sore throat in patients undergoing breast surgery. Physicians should consider this impact on clinical practice to optimize patient outcomes. Additional studies with a larger sample size are warranted to further explore this impact.

## Introduction

A common complication of anesthesia following endotracheal tube (ETT) placement is a sore throat, which can lead to dissatisfaction after surgery and delay a patient's return to normal activities. The incidence is highest after tracheal intubation and varies from 14.4% to 50% [1]. Many factors can contribute to postoperative sore throat, and the incidence can vary with airway management [2]. Safe anesthesia is a multidimensional anesthetic service provision that satisfies the demand for patient care and minimizes associated complications [3]. Good anesthetic care aims to achieve a secure airway and ventilate the lungs in a proper physiological manner. A safe and secure airway is achieved by ETT and laryngeal mask airway, primarily performed by an anesthetist or anesthesiologist. The ETT is made of polyvinylchloride and has a cuff at the distal end, which is inflated after being correctly placed in the trachea.

Although a postoperative sore throat is a minor problem, controlling it imposes a significant impact on a patient's comfort during the postoperative period [4]. The appropriate size of ETT plays a major role in preventing postoperative sore throat; however, there have been few studies regarding ETT size in the Pakistani population. Additionally, there are no published data on the frequency of this complication in cancer patients in our population. Therefore, the goal of this study was to compare the effect of the size of ETTs (6.5 mm and 7.5 mm) on postoperative sore throat in the local cancer population, which was helpful to improve the postoperative outcome in these patients.

Hypothesis

There is a difference in the frequency of postoperative sore throat in patients undergoing breast surgery with the 6.5-mm ETT compared to the larger 7.5-mm ETT.

## Materials and methods

This randomized controlled trial was conducted at the Shaukat Khanum Memorial Cancer Hospital and Research Center for a period of six months. A sample size of 110 cases (55 in each group) was calculated with an 80% power of test and a 5% level of significance. A nonprobability consecutive sampling technique was used. American Society of Anesthesiology (ASA) class I and II patients undergoing breast cancer in the age group of >18 to 65 years were included. Exclusion criteria were the use of succinylcholine, anesthesia with rapid sequence induction (e.g., applied cricoid pressure to prevent the risk of aspiration), patients having ongoing respiratory tract infection assessed by history and examination, and patients with a history of deep vein thrombosis.

After approval from the Shaukat Khanum Memorial Cancer Hospital and Research Center ethical committee and obtaining informed consent from the patients, a total of 110 patients selected from the elective surgery list fulfilling the inclusion criteria using a lottery method were included in the study. Standard monitoring was applied to all the patients preoperatively. General anesthesia was induced with intravenous 1.5 mg/kg propofol and 0.5/kg atracurium for muscle relaxation. After intravenous muscle relaxant, direct laryngoscopy was performed by an anesthetist (with more than six months' experience) using Macintosh laryngoscope blade no. 3. Group A patients were intubated with 6.5-mm ETT and group B patients with 7.5-mm ETT. After intubation, anesthesia was maintained by routine inhalation agent isoflurane or sevoflurane. After completion of the surgical procedure, a residual neuromuscular block was antagonized with 2.5 mg of neo-pyrolite intravenously. All patients were carefully extubated in the operating room when filling extubation criteria. Any concerns of sore throat were recorded at 24 hours postoperatively. All relevant data were noted on the prescribed pro forma. Sore throat was diagnosed by asking the patients. All the patients were females, and therefore a standard ETT of 7.5 mm was used for anesthesia.

Mean and standard deviation for age, body mass index (BMI), and four-point scale scores were calculated. Frequency and percentage for sore throat at 24 hours postoperatively were calculated. The two groups were compared for frequency using a chi-square test with p < 0.05 as the level of significance. Data were stratified for age and BMI to address the effect modifier. Poststratification chi-square test was applied to check the significance with p < 0.05 enabled as significant. All variables were analyzed using IBM SPSS Statistics for Windows, Version 20.0. (IBM Corp., Armonk, NY, USA).

## Results

A total of 110 patients, 55 from each group, selected from the elective surgery list fulfilling the inclusion criteria were included in the study. The mean age was 46.6 ± 13.2 years, with a minimum and maximum of 18 and 65 years, respectively. The mean BMI kg/m^2^ was 29.50 ± 07.12 kg/m^2^, with a minimum and maximum of 19 kg/m^2^ and 38 kg/m^2^, respectively. The mean four-point scale was 2.98 ± 1.3 (Table 1).

**Table 1:** Baseline characteristics of patients Abbreviation: ETT, endotracheal tube

Variable Categories	Total, N (%)
Age (years)	
Mean ± standard deviation	47 ± 13
Age groups (years)	
18-40	27 (24.55)
Above 40	83 (75.45)
Body mass index	
Mean ± standard deviation	29.50 ± 7.12
Body mass index groups	
Up to (20 kg/m^2^)	16 (14.55)
20-28 (kg/m^2^)	30 (27.33)
Above 28 (kg/m^2^)	64 (58.12)
Four-point scale score	
Mean ± standard deviation	2.98 ± 1.3
Sore throat (postoperatively within 24 hours)	
No	64 (57.6)
Yes	46 (42.4b)
ETT size	
Group A (6.5-mm ETT)	55 (50.0)
Group B (7.5-mm ETT )	55 (50.0)

The study results showed that 47 patients reported a sore throat, whereas 63 patients did not report a sore throat at 24 hours after surgery. Fourteen patients in group A were diagnosed with a postoperative sore throat at 24 hours, and 33 patients in group B were diagnosed with a sore throat. Chi-square was significant, and poststratification chi-square test was applied to compare sore throat at postoperative 24 hours among the groups for age and BMI (Table 2).

**Table 2:** Stratification comparison of postoperative sore throat Abbreviation: ETT, endotracheal tube

Variable Categories	Sore Throat	No Sore Throat	p-Value
ETT size (mm)	47 (42.73%)	63 (57.27%)	0.001
Group A (6.5-mm ETT)	14 (25.45)	41 (74.55)	
Group B (7.5-mm ETT)	33 (60.00)	22 (40.00)	
Age (18-40 years)	8 (29.63%)	19 (70.37%)	0.02
Group A (6.5-mm ETT)	2 (12.50)	14 (87.50)	
Group B (7.5-mm ETT)	6 (54.54)	5 (45.46)	
Age (41-65 years)	39 (46.99%)	44 (53.01)	0.005
Group A (6.5-mm ETT)	12 (30.77)	27 (69.23)	
Group B (7.5-mm ETT)	27 (61.36)	17 (38.64)	
Body mass index (up to 20 kg/m^2^)	7 (43.75%)	9 (56.25%)	0.01
Group A (6.5-mm ETT)	2 (20.00)	8 (80.00)	
Group B (7.5-mm ETT)	5 (83.33)	1 (16.67)	
Body mass index (21-28 kg/m^2^)	14 (46.67%)	16 (53.33%)	0.02
Group A (6.5-mm ETT)	4 (26.67)	11 (73.33)	
Group B (7.5-mm ETT)	10 (67.67)	5 (33.33)	
Body mass index (above 28 kg/m^2^)	26 (28.89%)	64 (71.11%)	0.03
Group A (6.5-mm ETT)	8 (26.67)	22 (73.33)	
Group B (7.5-mm ETT)	18 (52.94)	16 (47.05)	

## Discussion

Postoperative sore throat is among the most common anesthesia complications during the postoperative period. It impacts the well-being of patients after surgical procedures under general anesthesia. An anesthesia complication is a patient's physiological derangement or any unwanted effect related to anesthesia management.

Our patient demographics were similar to those of a similar study by Higgins et al., where the mean age of participants was 47 years, with a range of 16 to 85 years [1]. Jaensson et al. studied 100 intubated surgery patients, of which 82 were plastic surgery patients and 18 were undergoing ear, nose, and throat surgery [5]. They were randomly allocated to oral intubation with either ETT size 6.0 or 7.0. The incidence of postoperative sore throat in the postoperative care unit was greater in the ETT size 7.0 group (51%) than ETT size 6.9 (27.1%), which aligns with our findings.

Hu et al. carried out a meta-analysis in which three trials with 509 patients were included undergoing elective surgery [6]. Sizes of ETT were 6.0 and 7.0, and of 59 patients, 112 women developed postoperative sore throat in the postanesthesia care unit. The incidence of postoperative sore throat was low, with ETT size 6.0 (61%) compared to ETT size 7.0 (49%). Another study was conducted in Pakistan by Kadri et al. [7] to determine the frequency of postoperative sore throat after thyroidectomy at Isra University Teaching Hospital. Of 140 patients, postoperative sore throat was observed in 112 patients, and the frequency of postoperative sore throat was 22% with ETT size 7.0 and 78% with ETT size 8.0.

The incidence of postoperative sore throat has been documented from 24% to 90% or more in some situations [8]. This is due to multiple factors in the occurrence and severity of postoperative sore throat, as indicated by scholars [9]. The incidence of sore throat after tracheal intubation ranges from 14.4% to 50%; however, the use of laryngeal mask airway has an incidence of sore throat of 5.8% to 34%, indicating far fewer cases of sore throat when a mask is used compared to tracheal intubation for anesthesia [10]. The incidence was found to be greater in women because of the tight contact of the airway mucosa with airway equipment, yet other reports found no difference between men and women. One study identified no difference in the rate of sore throat occurrence among gender and lubrication of ETT [11]. 

Furthermore, the method of interview affects the reporting of a sore throat, that is, whether the questions are asked directly or indirectly. Studies have demonstrated that diclofenac premedication will not prevent the occurrence of sore throat [9]. However, another study conducted in Nepal demonstrated that preoperative ketamine gargling and magnesium medication significantly reduce the incidence of postoperative sore throat [12]. Dexamethasone showed promise in reducing the incidence of postoperative sore throat occurrence and severity [13].

Limitations

Our study was limited by its small sample size and short duration of anesthesia. A large sample size and long duration of anesthesia (lasting longer than six hours) are required for future studies to adequately confirm our findings.

## Conclusions

ETT sizes produced a significant difference in the frequency of postoperative sore throat in patients undergoing breast surgery. Physicians should consider this impact on clinical practice to optimize patient outcomes and contribute to improved patient quality of life. This study will help the patients especially cancer patients who are immunocompromised and fragile and for whom extra care must be made to avoid any complications. Due to such a high incidence of sore throat, we need further larger sample size trials in diverse populations that can help to identify factors preventing this side effect and need of other alternative methods to avoid it.
